# A quantitative atlas of Even-skipped and Hunchback expression in *Clogmia albipunctata* (Diptera: Psychodidae) blastoderm embryos

**DOI:** 10.1186/2041-9139-5-1

**Published:** 2014-01-07

**Authors:** Hilde Janssens, Ken Siggens, Damjan Cicin-Sain, Eva Jiménez-Guri, Marco Musy, Michael Akam, Johannes Jaeger

**Affiliations:** 1 EMBL/CRG Research Unit in Systems Biology, Centre de Regulació Genòmica (CRG), and Universitat Pompeu Fabra (UPF), Dr. Aiguader 88, 08003 Barcelona, Spain; 2 Department of Zoology, Downing Street, Cambridge CB2 3EJ UK

**Keywords:** *Clogmia albipunctata*, Non-drosophilid diptera, Non-model organism, Pattern formation, Comparative network analysis, Segmentation gene network, Hunchback, Even-skipped, Image bioinformatics, Quantitative expression data

## Abstract

**Background:**

Comparative studies of developmental processes are one of the main approaches to evolutionary developmental biology (evo-devo). Over recent years, there has been a shift of focus from the comparative study of particular regulatory genes to the level of whole gene networks. Reverse-engineering methods can be used to computationally reconstitute and analyze the function and dynamics of such networks. These methods require quantitative spatio-temporal expression data for model fitting. Obtaining such data in non-model organisms remains a major technical challenge, impeding the wider application of data-driven mathematical modeling to evo-devo.

**Results:**

We have raised antibodies against four segmentation gene products in the moth midge *Clogmia albipunctata*, a non-drosophilid dipteran species. We have used these antibodies to create a quantitative atlas of protein expression patterns for the gap gene *hunchback (hb)*, and the pair-rule gene *even-skipped (eve).* Our data reveal differences in the dynamics of Hb boundary positioning and Eve stripe formation between *C. albipunctata* and *Drosophila melanogaster*. Despite these differences, the overall relative spatial arrangement of Hb and Eve domains is remarkably conserved between these two distantly related dipteran species.

**Conclusions:**

We provide a proof of principle that it is possible to acquire quantitative gene expression data at high accuracy and spatio-temporal resolution in non-model organisms. Our quantitative data extend earlier qualitative studies of segmentation gene expression in *C. albipunctata,* and provide a starting point for comparative reverse-engineering studies of the evolutionary and developmental dynamics of the segmentation gene system.

## Background

One of the main approaches to evolutionary developmental biology (evo-devo) is the comparative study of developmental processes (for example, see [1,2]). Much of this work focuses on molecular (mainly transcriptional) regulatory networks [3-5]. Such studies reveal which aspects of development are conserved and which are more variable. This not only gives us insights into the evolutionary history of a developmental trait, but also enables us to better understand the functional principles of regulatory processes, and the constraints they impose on evolution (for example, see [6,7]).

The field of evo-devo is currently moving from comparative studies of gene regulation at the level of individual genes to more integrative approaches trying to compare the dynamics and function of entire regulatory networks (for example, see [4,5,8-19]). Only such a network-centered view can give us a rigorous understanding of important systems-level concepts, such as evolvability and robustness of developmental traits (for example, see [20-37]).

One particularly powerful approach to the integrative comparative study of development is the use of reverse-engineering approaches to reconstruct the structure and dynamics of regulatory networks across species [19]. This approach has a lot of potential since it does not require any genetic perturbations, which are often non-trivial to implement outside well established model organisms.

Reverse engineering of networks is based on quantitative measurement of gene expression patterns (reviewed in [19,38-41]). These data are then used to fit a gene network model. The resulting gene circuit solutions represent specific instances of a network that are capable of reproducing the observed patterns. These networks can then be analyzed to gain insights into the regulatory processes underlying observed gene expression or phenotypic traits (for example, see [42-49]).

In the context of pattern formation, it is especially important to preserve the spatio-temporal aspects of the data to be fit, since the focus is on the regulation of timing and spatial features of gene expression (such as domain boundaries). For this reason, we need to adapt methods for quantitative microscopy and image bioinformatics (for example, see [50,51]) to a non-model organism context. This poses a significant technical challenge which needs to be met if reverse-engineering methods are to be more widely applied in the context of evo-devo.

**Figure 1:**
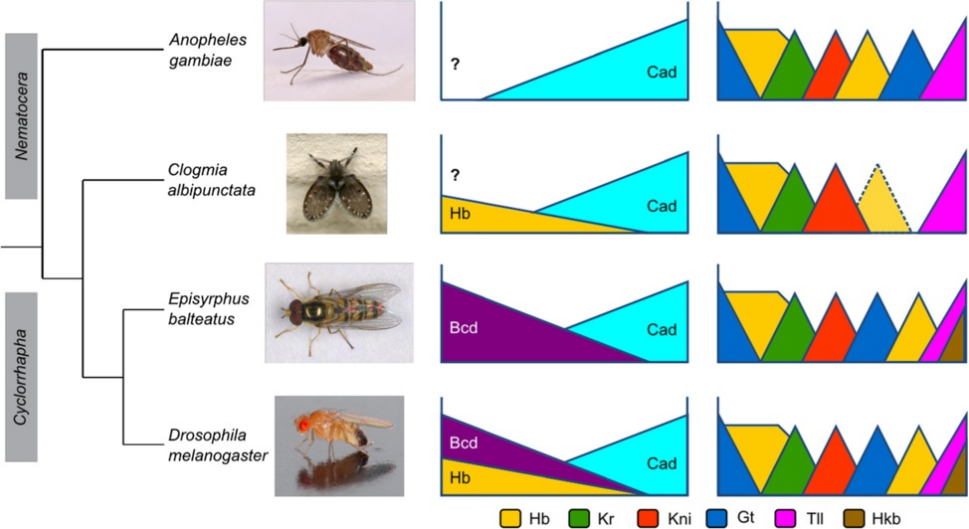
**Gap and maternal co-ordinate genes in different dipteran species.** This figure shows a simplified phylogeny of the Diptera. *Anopheles gambiae* (family: Culicidae) and *Clogmia albipunctata* (Psychodidae) both belong to the paraphyletic assemblage of the Nematocera. *Drosophila melanogaster* (Drosophilidae) and *Episyrphus balteatus* (Syrphidae) belong to the monophyletic cyclorrhaphan Brachycera (higher flies). The column of graphs on the left schematically shows the presence and expression patterns of maternal co-ordinate genes; graphs on the right show zygotically expressed gap gene domains (at the end of C14A, just before gastrulation; solid triangles), and the delayed appearance of the posterior Hb domain in *C. albipunctata* (after gastrulation; triangle with dashed outline). X-axes represent the antero-posterior embryonic axis: anterior is to the left, posterior to the right. Maternal co-ordinate gene products: Bcd, Bicoid; Hb, Hunchback; Cad, Caudal. Gap gene products: Hb, Hunchback; Kr, Krüppel; Kni, Knirps; Gt, Giant; Tll, Tailless; Hkb, Huckebein. Fly images from the Encyclopedia of Life (http://www.eol.org), photographers: *A. gambiae*, Muhammad Mahdi Karim; *C. albipunctata*, Gail; *D. melanogaster*, André Karwath; *E. balteatus*, Malcolm Storey. All images under creative commons license.

Here, we provide a proof of principle that quantitative measurements of expression patterns with high accuracy and spatio-temporal resolution are possible in a non-model organism. We have raised antibodies against four segmentation gene products in the nematoceran moth midge *Clogmia albipunctata* (family: Psychodidae; Figure 1). Gap and pair-rule gene expression in this species shows a number of interesting qualitative differences compared to the standard model for dipteran segmentation, the vinegar fly *Drosophila melanogaster.* In particular, expression of posterior gap domains is modified and pair-rule stripes are delayed in *C. albipunctata* compared to *D. melanogaster* (Figure 1; [52-54]). To investigate these differences in more detail, we use immunofluorescence combined with confocal scanning microscopy and a data processing pipeline adapted from *D. melanogaster*[50] to provide a quantitative atlas of protein expression patterns for the gap gene *hunchback (hb)*, and the pair-rule gene *even-skipped (eve).* We analyze these expression patterns with regard to their timing and spatial registration. Our results show that the dynamics of Hb boundary positioning and Eve stripe formation differ significantly between *C. albipunctata* and *D. melanogaster.* Anterior shifts in domain position as development proceeds are not only present, but much more pronounced in *C. albipunctata.* Despite this, the relative arrangement of the anterior Hb domain and the anterior stripes of Eve is largely conserved across the evolutionary distance between the two species. The quantitative dataset that we have produced provides a suitable starting point for future reverse-engineering approaches to study the mechanisms and dynamics of segmentation gene regulation in *C. albipunctata.*

## Methods

### Embryo collection and fixation

*C. albipunctata* culture and embryo collection methods have been described previously [54] (detailed protocols are available from the authors on request). Embryos were dissected from adult females, development was activated by osmotic shock, and embryos were left to develop until the desired stage on moist filter paper at 25°C. Blastoderm stage embryos were fixed at a number of time points of development up to a maximum of 8 hours after egg activation (see Results for details). Fixation was performed using a modification of a previously described procedure [54]. Following dechorionation in 50% bleach, and fixation in a 1:1 mixture of 8% formaldehyde in PBS/heptane for 25 minutes, the formaldehyde/heptane was removed. Heptane was added to the embryos followed by an equal volume of methanol (pre-cooled to −80°C), and this mixture was shaken for 20 to 30 seconds to fracture the vitelline layer. The heptane/methanol was removed and replaced with methanol. Embryos were transferred to a 10-ml syringe (fitted with a 24 g × 1 inch; 0.7 mm needle and with the syringe plunger removed) using additional methanol as required to aid the transfer of all of the embryos. With approximately 5 ml methanol/embryos in the syringe, the plunger was refitted, the embryos were dispersed by shaking so as not to be sitting in the outlet port of the syringe, and the embryos were then rapidly expelled through the needle into a 15 ml glass vial. The embryos were then transferred to a 1.5 ml microcentrifuge tube and washed three times with methanol. Devitellinized embryos were stored in methanol at −20°C.

### Antibody production

Polyclonal antisera were raised against *C. albipunctata* Hunchback (Calb-Hb), Giant (Calb-Gt), and Knirps-like (Calb-Knl; see [54] for gene nomenclature) proteins expressed by means of pET-DEST42 vector/cDNA constructs (Invitrogen, Life Technologies, Carlsbad, CA, USA) using procedures described previously [47]. Antibodies against Calb-Hb were raised in two rabbits using 100 μg protein per immunization injection (Eurogentec, Liège, Belgium). Antibodies against Calb-Gt and Calb-Knl were raised in two guinea pigs for each protein using 50 μg protein per immunization injection (Eurogentec, Liège, Belgium).

*C. albipunctata* has two paralogues of the *D. melanogaster even-skipped* gene (*Calb-eve1* and *Calb-eve2*) which are both expressed in very similar patterns during the blastoderm stage [53,54]. Polyclonal antiserum against *C. albipunctata* Even-skipped1 protein (Calb-Eve1) was raised as follows. A pET-DEST42 vector/Eve1 cDNA construct was produced. Exponentially growing bacterial cultures were induced with 0.5 mM isopropyl β-D_1-thiogalactopyranoside (IPTG) for 2 hours and bacterial pellets were harvested and stored at −20°C. Cleared lysate preparation and purification of the 6xHis-tagged Eve1 protein under denaturing conditions were carried out using Ni-nitrilotriacetic acid (Ni-NTA) agarose protocols (Qiagen, Venlo, Limburg, Netherlands). Briefly, cells were lyzed in 8 M urea lysis buffer (Buffer B, Qiagen) for 1 hour with constant mixing. Following centrifugation, the solubilized protein supernatant was added to Ni-NTA Agarose and mixed at a low speed on a rotary shaker for 1 hour to allow binding of the His-tag. Subsequent recovery, washing and elution steps were carried out using centrifugation for 5 seconds at 1000 g. Bound protein was eluted using Buffer E (8 M urea buffer pH 4.5, Qiagen). Finally, the purified Eve1 was dialyzed against deionized water then quantified and aliquoted for the immunization program. Antibodies were raised in two guinea pigs using 50 μg protein per immunization injection (Eurogentec, Liège, Belgium).

### Antibody staining

#### Immunofluorescent protocol

Staged embryos were stained with antisera against Calb-Hb and Calb-Eve1. Briefly, embryos were rehydrated through graded methanol/PBT washes (PBT is PBS, 0.1% Tween) then washed 2x30 minutes in PBT. Embryos were incubated for 2x60 minutes in PBTB blocking buffer (PBTB is PBT plus Western Blocking Reagent, Roche, Basel, Switzerland). Primary antibodies were pre-absorbed onto *D. melanogaster* 0–24 hour fixed embryos overnight at 4°C. Rabbit anti-Hb (serum SK4433) was preabsorbed at 1:100 dilution in PBT; guinea pig anti-Eve1 (serum SKC044) was preabsorbed at 1:25 dilution in PBT. Primary antibody stainings were done in 800 μl PBTB + 100 μl of each preabsorbed antibody (rabbit anti-Hb, guinea pig anti-Eve1) at 4°C overnight. Embryos were then washed 4×20 minutes in PBT followed by 2×30 minutes in PBTB. Secondary antibody incubations were done in 1 ml PBTB/antibody for 2 hours at room temperature. Secondary antibodies were anti-rabbit-Alexa647 and anti-guinea pig-Alexa555 at a dilution of 1:4000 (Molecular Probes, Life Technologies, Carlsbad, CA, USA). Embryos were washed 2×15 minutes in PBT and then counterstained for 10 minutes with Hoechst 34580 (Molecular Probes) at a dilution of 1:1000 in PBT. Embryos were washed 2×1 hour in PBT at room temperature, then washed in PBT overnight at 4°C; finally, the embryos were equilibrated overnight in 1,4-diazabicyclo[2.2.2]octane (DABCO) mounting solution at 4°C (5% DABCO in 90% glycerol/PBS pH 8) prior to slide preparation.

#### Colorimetric (enzymatic) protocol

This assay is based on the following modifications of the immunofluorescent antibody staining procedure. Anti-guinea pig-AP conjugate (The Jackson Laboratory, Bar Harbor, ME, USA) was used as the secondary antibody. Detection was carried out using NBT/BCIP (Roche, Basel, Switzerland). Signal development was allowed to proceed at room temperature until patterning was visible and was stopped by washing with PBT; embryos were mounted as for confocal procedure.

### Quantitative gene expression data

Image acquisition and data processing for *C. albipunctata* embryos stained using antisera against Calb-Hb and Calb-Eve1 was performed using a quantification pipeline involving the following steps: (1) images were acquired using a 20× water-immersion objective on a SP5 confocal microscope (Leica Microsystems, Wetzlar, Germany) as described previously [47]; (2) dorso-ventral (D-V) orientation was determined based on membrane morphology [55], and slanting of Eve stripes, as described in Results; (3) image segmentation was performed to identify nuclei and measure fluorescence intensity per nucleus as described [50,56,57]; (4) embryos were sorted into time classes as described below; (5) non-specific background staining was removed as described [50,57,58]; (6) a strip along the lateral midline - covering 10% of the embryo’s height (D-V) - was extracted using a previously published graphical user interface [51]; (7) data registration was performed by spline approximation [50,57,59] using the BREReA software (http://urchin.spbcas.ru/downloads/BREReA/BREReA.htm; successor of GCPReg [60]); (8) data integration was performed by collecting data points into 100 bins along the antero-posterior (A-P) axis and then averaging individual profiles for each gene and time class [50,57]; (9) integrated data were smoothened by applying a Gaussian filter with a kernel width of three nuclei; and (10) expression levels were scaled to facilitate comparison between datasets.

Positions of Calb-Eve protein stripes were calculated as described previously [61] by approximating the expression data with quadratic splines [59]. Positions of the posterior boundaries of the anterior Hb domain were calculated by extracting points of half-maximum fluorescence intensity using fast dyadic wavelets [59].

### Time classification

Embryos were assigned to blastoderm cleavage cycles 10-14A (C10-C14A, C14A is the part of C14 that occurs before gastrulation [62]) based on the observed number of nuclei and nuclear density [55]. C14A embryos were further classified into 8 time classes (T1-T8) based on visual inspection of Calb-Eve protein staining. The assignment of ambiguous cases was corrected using membrane morphology whenever possible (based on the morphological staging scheme described in [55]).

Because time classification of embryos could be affected by observer bias, we developed an algorithm to verify and confirm the staging of embryos for time classes T5-T8. This involves searching for clusters of embryos which show Calb-Eve expression profiles of similar shape in a multidimensional space with a suitable definition of a clustering metric. The algorithm follows two basic steps. All combinations of embryos that constitute our dataset are fitted against each other, two by two, and the corresponding probability of X^2^_*v*_ is used to define a relative distance between embryos. The second step is to build clusters of close-by embryos. We start by considering individual embryos as clusters of one object. A hierarchical clustering method was then used: at each iteration, the two closest clusters are found and merged into a single cluster for which the distances to all the other clusters are recalculated as the mean of the initial two. The process can be stopped at any arbitrary number of desired clusters (eight in our case). Additional signatures can be taken into consideration to improve algorithm performance and further refine the clustering: the relative ratio of intensities for stripes 1 and 6, and/or the ratio of their widths. These two additional factors can be merged into one single variable by a classic Principal Component Analysis method. Considering these additional signatures resulted in a final number of four clusters, which correspond to the previously established time classes T5-T8.

### Statistical analysis of gene expression data

We applied a two-sided Welch *t*-test (both on unranked and ranked data) to calculate if total Eve domain width differs between *C. albipunctata* and *D. melanogaster.* Domain widths for testing were measured from the peak of stripe 1 to the peak of stripe 6 in *C. albipunctata*, and from the peak of stripe 1 to the peak of stripe 7 in *D. melanogaster*.

## Results and discussion

### Polyclonal antisera against *C. albipunctata* segmentation proteins

**Figure 2:**
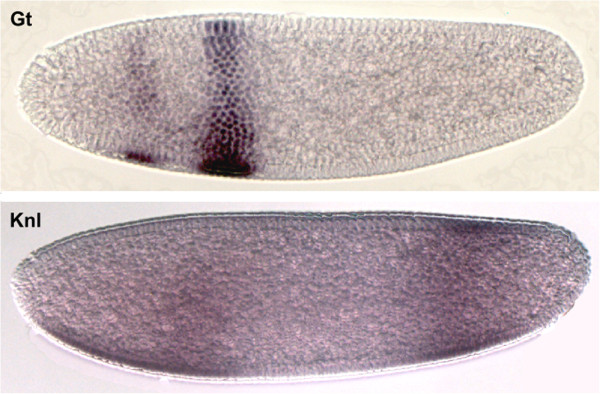
**Colorimetric (enzymatic) antibody stains against Giant (Gt) and Knirps-like (Knl) in *C. albipunctata *embryos.** Blastoderm-stage embryos were stained with antibodies against Calb-Gt protein (antibody SKC037, upper panel) and against Calb-Knl protein (antibody SKC039, lower panel). Lateral embryo images are shown where anterior is to the left, dorsal is up.

We raised antibodies against the following proteins in *C. albipunctata*: Calb-Gt, Calb-Knl, Calb-Hb, and Calb-Eve1 (see Additional file 1: Table S1). Test stains using a colorimetric (enzymatic) experimental protocol show that all these antisera produce staining patterns consistent with mRNA expression of the corresponding genes [54]. Example stains for Calb-Gt and Calb-Knl are shown in Figure 2. For Calb-Hb and Calb-Eve1, see our detailed analysis based on immunofluorescent staining protocols below.

### Immunofluorescent staining in *C. albipunctata* embryos

**Figure 3:**
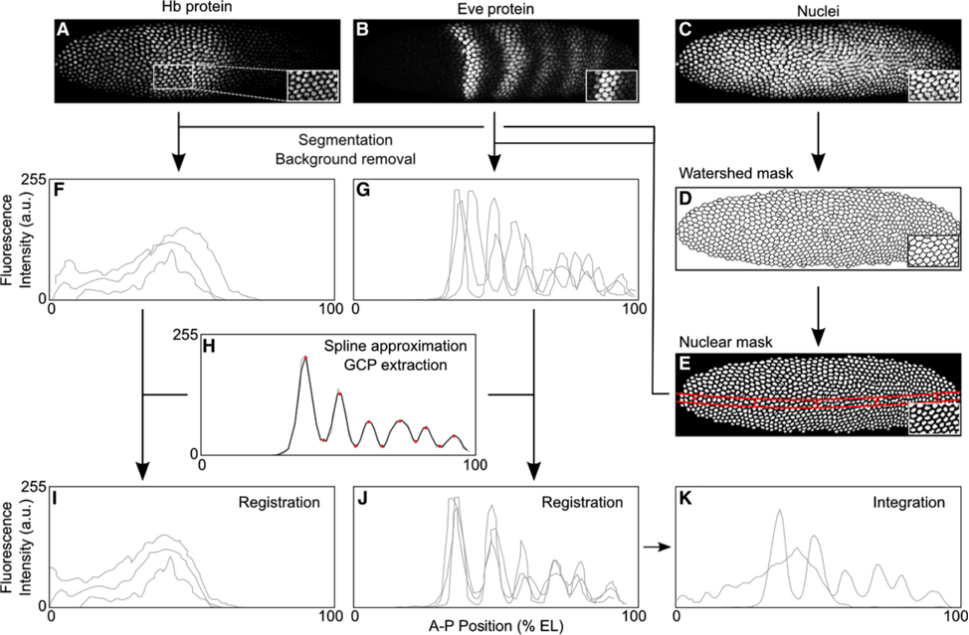
**Summary of image processing.** Upper panels show scanning confocal microscopy images of an example embryo stained against Hb protein **(A)**, against Eve protein **(B)**, and with a nuclear counterstain **(C)**. Insets show magnified details at the position indicated by a white square in **(A)**. Nuclear counterstains **(C)** are used to generate binary watershed masks **(D)** and nuclear masks **(E)** for image segmentation. Individual expression profiles after image segmentation and background removal are shown for Hb **(F)** and Eve **(G)**. These profiles are extracted from a 10% strip along the midline of the embryo, as shown by red lines in **(E)**. Data registration is performed by approximating the Eve pattern using quadratic splines **(H)**: Ground Control Points (GCPs, red dots in **H**) are extracted and used as the basis for an affine coordinate transformation which minimizes embryo-to-embryo variation in their position across the dataset **(J)**. The same transformation is applied to the Hb profile **(I)**. Finally, individual expression profiles belonging to the same time point are classified into 100 bins along the antero-posterior (A-P) axis, and data points within bin are averaged to yield an integrated dataset for both Hb and Eve **(K)**. See Methods for details. a.u., arbitrary units; EL, egg length.

We previously established an image acquisition and quantification method to generate gene expression datasets with high temporal and spatial resolution in *D. melanogaster* embryos (reviewed in [50]). Generating the same type of high-quality spatio-temporal data in a non-model organism such as *C. albipunctata* turns out to be quite challenging; in this species, embryos vary in shape, embryo morphology is less robust towards experimental procedures, antibody stainings are noisy, and the presence of the extraembryonic tissues (amnion and serosa) causes differential staining patterns along the D-V axis. For these reasons, considerable adaptation and optimization of embryo-fixation, dechorionation/devitellination, as well as immunofluorescent antibody staining protocols was required to produce data of acceptable quality (see also Methods). Using these adapted protocols, we stained a large number of *C. albipunctata* blastoderm embryos against Hb and Eve proteins (Figure 3A,B; see also Additional file 2: Table S2) while nuclei were visualized using a Hoechst34580 (Molecular Probes) counterstain (Figure 3C). For each embryo, we acquired images of the two data channels, and the nuclear channel (all at two different z-positions to capture as many blastoderm nuclei as possible) using confocal scanning microscopy. In addition, we imaged embryo morphology using differential interference contrast (DIC; not shown).

We do not know whether the Calb-Eve1 antiserum also binds to the product of the *eve2* gene, which is very similar in sequence and shows an expression pattern similar to that of *eve1*[53,54]. Therefore, we refer to these stains simply as ‘Eve’ below.

### Embryo orientation

In a majority of embryos, membrane morphology [55] and/or the presence of extraembryonic tissues allowed us to determine the D-V orientation of the embryo. In case those features were not distinct enough to orient the embryos, we used the slanting of Eve stripes 1 and 2 as indicators, since these stripes slant towards the posterior in a consistent manner. In some of the younger embryos (pre-T3/T4), D-V alignment is harder to determine, but also less crucial due to the absence of significant D-V asymmetry in Hb and Eve expression patterns.

### Image processing and data quantification

Nuclear images were processed using watershed-based image segmentation to generate a binary nuclear mask (Figure 3D,E), where each nucleus is clearly separated from its neighbors [56]. We then extracted data from a 10% strip on the lateral side of the embryo: this region is determined manually as described in [51] (red lines in Figure 3E). This allows us to avoid measuring gene expression in the extraembryonic anlage, and to deal with the large variability in embryo shape. Next, non-specific background staining was removed, and data were registered in order to eliminate embryo-to-embryo variation, which is crucial for averaging data per gene and time class (Figure 3H–K, see Methods for details). This resulted in an integrated atlas of Hb and Eve expression in *C. albipunctata* based on a total of 484 selected embryos (Additional file 2: Table S2). This dataset is currently not hosted on any public database, but is available from the authors upon request.

### Time classification

In a parallel effort to this study, we have carefully characterized *C. albipunctata* development and morphogenesis using live DIC imaging [55]. This work revealed that, just as in *D. melanogaster*, there are 14 cleavage cycles (C1-C14A) before gastrulation. Embryos can be assigned to separate cleavage cycles based on nuclear density and membrane morphology. Earlier work using quantitative expression data in *D. melanogaster* further subdivided C14A into eight separate time classes [50]. To facilitate comparisons between the two species, we also divide C14A in *C. albipunctata* into eight time classes (T1-T8; see also [55]). Assignment of embryos to these time classes is based on visual inspection of Eve expression pattern and membrane morphology, verified by cluster analysis, as described in Methods. We detect a positive correlation between assigned time class and age of embryos at fixation time (see Additional file 3: Table S3), further supporting our pattern-based staging scheme.

### Analysis of Eve protein expression in *C. albipunctata*

Previous studies using antibody stains or *in situ* hybridization of *Calb-eve* protein or mRNA revealed a heterochronic shift in the formation of Eve stripe 7, as only stripes 1 to 6 can be detected before gastrulation [52-55]. In addition, there seems to be a general delay in the formation of posterior *eve* stripes [55]. Here, we extend these earlier qualitative studies with a quantitative analysis of Eve expression at the protein level.

**Figure 4:**
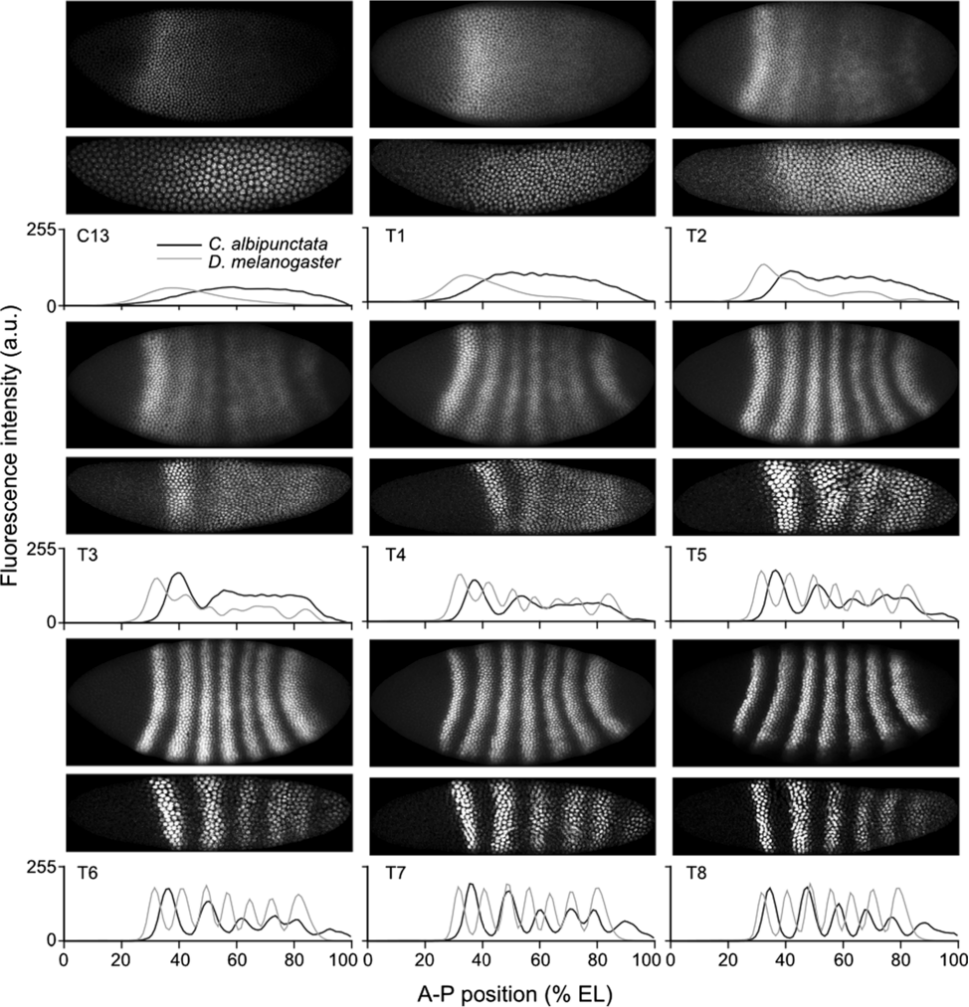
**Comparison of quantitative Eve protein expression patterns between *Clogmia albipunctata *and *Drosophila melanogaster*.** Representative embryo images showing Eve protein expression in *D. melanogaster* (upper rows) and *C. albipunctata* (lower rows) are shown for each time class in C13 and C14A (T1-T8). Lateral views; anterior is to the left, dorsal up. The graphs show corresponding integrated (averaged) expression patterns for each time class. *C. albipunctata* data are shown in black, *D. melanogaster* data in grey. Expression levels (vertical axes) are in relative units ranging from 0 to 255. Horizontal axes represent anterior-posterior (A-P) position in percent (where 0% is the anterior pole). To facilitate pattern comparison, expression levels in *C. albipunctata* were scaled in order to match expression peaks between the two species. a.u., arbitrary units; EL, egg length.

*C. albipunctata eve* mRNA expression starts around C12 [55] or C13 [54]. The earliest clearly detectable Eve protein expression is visible at C13 in a broad region from approximately 40% A-P position (where 0% is the anterior pole) to the posterior tip of the embryo (Figure 4). In *D. melanogaster*, Eve protein is detected as early as C12 - in a smaller domain, never reaching the posterior of the embryo [63].

The dynamics of Eve stripe formation differs significantly between the two species (Figure 4; see also [55]). In *D. melanogaster*, the initial broad Eve protein expression pattern becomes divided into two sub-domains: the anterior one later splits into stripes 1-2-3, and the posterior one into stripes 4-5-6. Stripe 7 arises *de novo* - that is, as a separate new domain [63]. In contrast, Eve stripes in *C. albipunctata* generally form by budding off from the initial broad domain, one by one in a roughly anterior-to-posterior sequence. The only exception to this rule is stripe 6, which forms *de novo.* All stripes resolve relatively late: stripe 1 forms first (T2), followed by stripe 2 (T3/T4), stripe 3 (T5) and then stripe 6 (T5/T6). As in *D. melanogaster,* the last stripes to become resolved are stripes 4 and 5, whose inter-stripe domain clears only around T7. The above indicates a general delay in stripe formation compared to *D. melanogaster* where all 7 Eve stripes are clearly visible by T4 [63]. Moreover, in contrast to *D. melanogaster*, Eve stripe 6 in *C. albipunctata* forms at the posterior pole of the embryo, its posterior boundary only retracting from the pole towards the end of T8. This creates an expression-free posterior region within which stripe 7 will form *de novo* after the onset of gastrulation [52].

While stripe formation differs markedly between the two species, stripe maturation is quite similar. In both species, inter-stripe minima further clear and deepen, expression levels rise, stripes sharpen, and shift to the anterior of the embryo after their initial formation (see below, and [63]).

Our quantitative results on the dynamics of Eve protein stripe formation and maturation are consistent with those revealed by earlier studies at the level of *eve* mRNA [54,55].

### Analysis of Hb protein expression in *C. albipunctata*

Two qualitative differences in *hb* expression between *C. albipunctata* and *D. melanogaster* had been reported previously [52,54]: (1) *C. albipunctata* lacks a posterior *hb* domain before gastrulation; and (2) *hb* is expressed in the antero-dorsal anlage of the serosa, an extraembryonic tissue. Here, we extend these earlier qualitative studies at the level of *hb* mRNA with a quantitative analysis of Hb protein expression.

**Figure 5:**
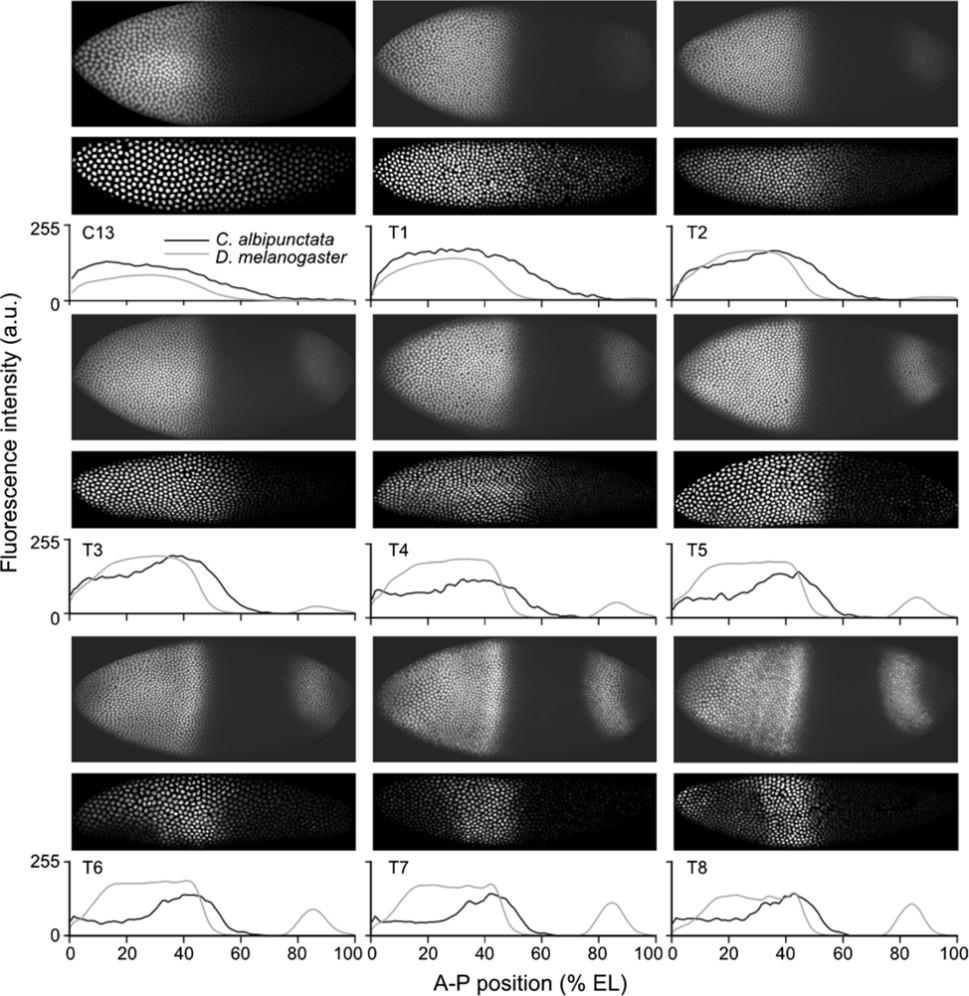
**Comparison of quantitative Hb protein expression patterns between *Clogmia albipunctata *and *Drosophila melanogaster*.** Representative embryo images showing Hb protein expression in *D. melanogaster* (upper rows) and *C. albipunctata* (lower rows) are shown for each time class in C13 and C14A (T1-T8). Lateral views; anterior is to the left, dorsal up. The graphs show corresponding integrated (averaged) expression patterns for each time class. *C. albipunctata* data are shown in black, *D. melanogaster* data in grey. Axes as in Figure 4. To facilitate pattern comparison, expression levels in *C. albipunctata* were scaled in order to match expression peaks between the two species. a.u., arbitrary units; EL, egg length.

In *D. melanogaster*, maternal Hb protein forms an A-P gradient around C10 [63,64]. In *C. albipunctata*, Hb protein can be detected slightly later: in C12 embryos, we can occasionally detect ubiquitous Hb staining, or the onset of a shallow A-P gradient. The first clear zygotic Hb protein expression is detected in C13 (Figure 5), consistent with the reported timing of mRNA expression [54]. While García-Solache and colleagues [54] reported an early homogenous *hb* mRNA expression throughout the anterior 60% of the embryo, Hb protein extends more posteriorly, reaching 75 to 80% A-P position (Figure 5). During C14A, Hb protein clears from the posterior of the embryo, and the posterior boundary of the Hb protein domain sharpens and shifts anteriorly to about 60% A-P position (compared to about 50% in the mRNA data [54]). Such an anterior shift is never observed in *D. melanogaster* (see also below). The Hb domain reaches its maximum expression level at T3, compared to T4 in *D. melanogaster* (Figure 5; see also [63]).

Towards the end of C14A, the anterior Hb domain of *D. melanogaster* splits into several parts, with the strongest expression at the most posterior end of the original domain (the parasegment-4 or PS4 stripe; [63]). Similarly, the anterior *hb* mRNA domain in *C. albipunctata* resolves into multiple bands at that stage [54]. In contrast, Hb protein expression seems to be less complex: expression decreases anteriorly, resulting in retraction from the anterior pole (Figure 5, T5-T8). This retraction only occurs in the embryonic anlage, while Hb remains expressed in the antero-dorsal anlage of the extraembryonic tissues (Figure 5, especially T6 and T8, the effect is less clear, but still visible, in the embryo shown for T7). The anterior Hb protein domain never splits into sub-domains, showing broad expression from 25 to 52% A-P position in the embryonic anlage. The only slightly non-uniform expression feature in this domain is a plateau of lower expression levels towards the anterior (Figure 5).

In summary, expression of the anterior domain of Hb is both more dynamic and less complex in *C. albipunctata* than in *D. melanogaster*: while the posterior boundary of Hb shifts significantly to the anterior, the domain never diversifies and splits into sub-domains during the late blastoderm stage.

### *C. albipunctata* Hb and Eve expression domains shift anteriorly over time

**Figure 6:**
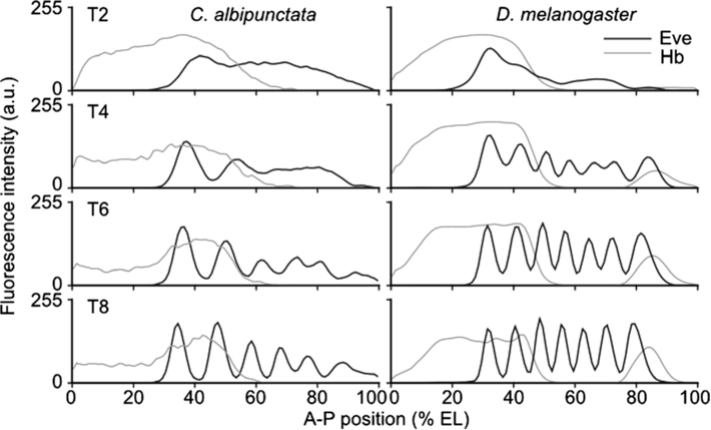
**Relative positioning of Eve and Hb protein expression domains is conserved in *Clogmia albipunctata *compared to *Drosophila melanogaster *embryos.** Integrated quantified expression patterns of Eve (black) and Hb (grey) are shown for *C. albipunctata* (left column) and *D. melanogaster* (right column) at time classes T2, T4, T6 and T8. Axes as in Figure 4. Scaling of expression levels as in Figures 4 and 5 to facilitate comparison. See text for details. a.u., arbitrary units; EL, egg length.

**Figure 7:**
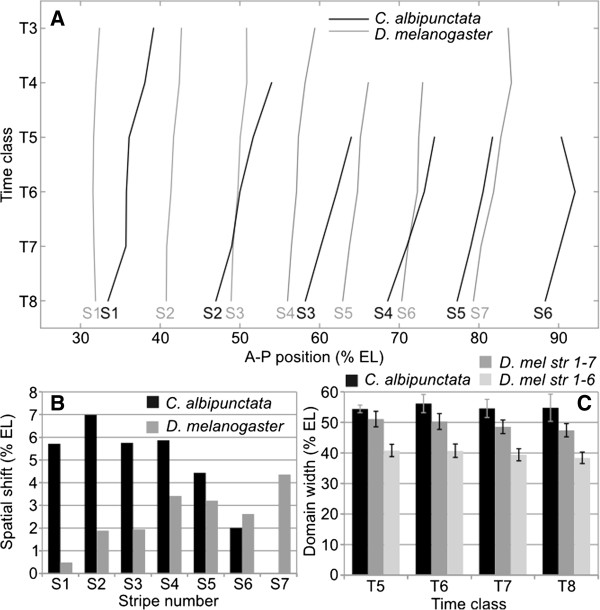
**Eve protein expression dynamics and variability in *Clogmia albipunctata *compared to *Drosophila melanogaster*. (A)** A time-space plot indicating the shifting positions of Eve stripes 1 to 7 (S1-S7) from the appearance of each stripe until T8. Lines represent linear interpolation between measured time points. *C. albipunctata* data are shown in black, *D. melanogaster* data in grey. Vertical axis indicates time (flowing downward); horizontal axis indicates percent antero-posterior (A-P) position (where 0% is the anterior pole). The graph only shows the region between 25 and 95% A-P position. **(B)** The total extent of shifts in peak positions (in % egg length, EL) for each stripe (S1-S7) in *C. albipunctata* (black) and *D. melanogaster* (grey) measured from the appearance of each stripe until the end of T8. **(C)** Shows the width of the entire region expressing Eve protein (in % EL) measured from the peak of stripe 1 to the peak of stripe 6 (*C. albipunctata*, black; *D. melanogaster,* light grey) or 7 (*D. melanogaster* only, dark grey) across time classes in C14A (T5-T8). Error bars represent one standard deviation from the mean. The difference in width between the *C. albipunctata* Eve domain (peak of stripe 1 to stripe 6), and that of *D. melanogaster* (stripe 1 to stripe 7) is statistically significant (*P* < 0.0005).

As reported for *eve* mRNA [54,55], each Eve protein stripe is located more posteriorly in *C. albipunctata* than its equivalent in *D. melanogaster* (Figure 6, Figure 7A, Additional file 4: Table S4). In fact, stripes 3–6 are initially located more than one stripe’s width more posterior than their *D. melanogaster* counterparts (Figure 7A). This raises the question whether this posterior displacement could involve reduced anterior domain shifts in *C. albipunctata* compared to *D. melanogaster*[63]. Our data reveal that this is not the case. On average, the shift of Eve stripes towards the anterior is twice as strong in *C. albipunctata* (5.31 nuclei) as in *D. melanogaster* (2.56 nuclei; see Additional file 4: Table S4), and every Eve stripe (except stripe 6) shifts over a larger number of nuclei in *C. albipunctata* compared to their *D. melanogaster* counterparts (Figure 7A,B; Additional file 4: Table S4). The fact that the shift mechanism in *D. melanogaster* does not depend on diffusion and hence nuclear spacing [43] makes it unlikely that this difference is caused by the reduced nuclear density observed in *C. albipunctata*[55]. A more complicated underlying cause for this difference is further suggested by the observation that *C. albipunctata* stripes 2 and 4 even “overtake” a *D. melanogaster* stripe (stripes 3 and 6, respectively; see Figure 7A). Interestingly, whereas in *D. melanogaster* the posterior Eve stripes shift more than the anterior stripes, *C. albipunctata* Eve stripes show the reverse (Figure 7A,B; Additional file 4: Table S4).

In *D. melanogaster*, the posterior boundary of the anterior Hb domain remains at a constant position over time [63]. In contrast, this boundary shifts markedly to the anterior in *C. albipunctata* mRNA *in situ* stains [54]. Our measurements confirm this result at the protein level: the posterior boundary of anterior Hb shows a small shift of about 2.15% egg length between T3 and T8.

In *D. melanogaster*, the anterior Hb domain overlaps with Eve stripes 1 and 2 from the time point on when they become detectable (Figure 6). In contrast, the relative domain positions of Eve and Hb are much more dynamic in *C. albipunctata* (Figure 6): during early C14A, the anterior Hb domain only overlaps with Eve stripe 1. Since Eve stripes 1 and 2 shift further anterior than the posterior boundary of the anterior Hb domain (5.72 and 6.99 versus 2.15% EL, respectively; see Additional file 4: Table S4), Eve stripe 2 eventually passes the position of the Hb border resulting in a similar relative arrangement of domains as seen in *D. melanogaster* by the end of C14A.

Analysis of movies based on live DIC imaging of early development in *C. albipunctata* show that nuclei do not move at all during blastoderm-stage interphases [55]. This implies that the observed anterior domain shifts are due to dynamic gene regulatory interactions, rather than physical relocation of nuclei, a conclusion also found in *D. melanogaster*[44,65].

Finally, we have measured the total width of the Eve domain in both species at different time points to investigate whether stripe shifts and refinement lead to compaction of the Eve-expression regions as seen in *D. melanogaster*[63]. Our measurements indicate that the Eve domain does not contract in *C. albipunctata*, but instead retains its total width as expression shifts anteriorly (Figure 7C; Additional file 5: Table S5). This is due to the increased shift in anterior, compared to posterior stripe positions (Figure 7A). Interestingly, the total relative width of Eve expression - measured as the distance between the peaks of stripe 1 and 6 - is wider in *C. albipunctata* both compared to the distances between stripes 1/6 and 1/7 in *D. melanogaster* (Figure 7C).

## Conclusions

In this paper, we present a quantitative analysis of the spatio-temporal protein expression patterns of two segmentation genes - the gap gene *hb*, and the pair-rule gene *eve* - in a non-drosophilid dipteran, the moth midge *C. albipunctata*. Our work extends earlier qualitative studies of segmentation gene expression in this species [52-55]. We confirm that the formation of Eve stripes is delayed in *C. albipunctata* compared to *D. melanogaster*, and that gap and pair-rule patterns shift anteriorly over time in this species. In addition, we show that domain shifts are much larger than those in *D. melanogaster*, and describe the precise dynamics by which the relative arrangement of the Hb domains with anterior Eve stripes is established. To our knowledge, no gene expression patterns have been studied with such accuracy and spatio-temporal resolution in any organism outside well established experimental model systems.

Our work provides a proof of principle that such detailed and systematic quantitative analyses of spatio-temporal gene expression are feasible in non-model organisms. Our data provide a powerful resource for reverse-engineering developmental gene regulatory networks [42-49]. We expect that increased availability of such data will promote the use of reverse-engineering methods for the comparative study of the evolution of developmental processes [19]. Ultimately, the computational reconstitution and analysis of developmental gene regulatory networks will lead to a much more systematic and quantitative understanding of the non-linear map from genotype to phenotype, tackling a central problem in current evo-devo [34,66].

## Abbreviations

D-V: Dorso-ventral; A-P: Antero-posterior; EL: Egg length; Calb-Gt: *C. albipunctata* Giant; Calb-Hb: *C. albipunctata* Hunchback; Calb-Knl: *C. albipunctata* Knirps-like; Calb-Eve: *C. albipunctata* Even-skipped; DIC: Differential interference contrast; PBS: Phosphate-buffered saline; evo-devo: Evolutionary developmental biology; a.u.: Arbitrary units.

## Competing interests

The authors declare that they have no competing interests.

## Authors’ contributions

HJ performed data processing and analysis. KS performed the experimental and microscopy work. DCS developed software tools for data processing and analysis. EJG contributed to image processing and helped with time classification of embryos. MM developed the clustering algorithm for time classification. MA and JJ designed the study. HJ and JJ wrote the manuscript. All authors read and approved the final manuscript.

## Supplementary Material

Additional file 1: Table S1 Polyclonal antisera against *C. albipunctata* Segmentation Proteins. This table summarizes the polyclonal antisera we have raised for this study. ‘Protein’ indicates the *C. albipunctata* gene products against which antibodies were raised. Gene nomenclature as in García-Solache and colleagues, *Dev Biol* 2010, 344:308–318. ‘Species’ indicates which animal was used for the immunization protocol. ‘Serum’ identifies distinct immunization reactions (two injections into different animals per gene product). ‘Rating’: ‘+++’ indicates minimal background and clear signal; ‘++’ indicates that higher concentrations of antibody are needed and background is significant; ‘+’ indicates a recognizable staining pattern, but low signal-to-noise ratio. Rating scheme equivalent to that used in Kosman and colleagues, *Dev Genes Evol* 1998, 208:290–294. Click here for file

Additional file 2: Table S2 Number of embryo expression profiles used for data quantification, per gene and time class. Click here for file

Additional file 3: Table S3 Number of embryos per time class according to physiological age. This table plots the number of embryos per assigned time class (C10-C13, C14A: T1-T8) versus the time of fixation (in hours:minutes after egg activation). See Methods in the main text for details on egg activation, embryo fixation, and assignment of embryos to time classes. Click here for file

Additional file 4: Table S4 Average positions and shifts of Eve stripe peaks in *C. albipunctata* (*Ca*) and *D. melanogaster* (*Dm*) embryos. This table shows the position (in % position along the A-P axis, where 0% is the anterior pole) of the point of maximum intensity within each visible Eve stripe from T3 to T8. The bottom row shows the extent (in % embryo length) of anterior temporal shifts in peak position for each detectable Eve stripe, calculated from the time of stripe appearance to T8. Positions and shifts are calculated from integrated data. See Methods in the main text for details. Click here for file

Additional file 5: Table S5 Width of the Eve domain in *C. albipunctata* and *D. melanogaster* embryos. Data shown for time classes T5-T8. Total domain widths (in % egg length) are calculated - from integrated data - as reaching from the peak of Eve stripe 1 to the peak of Eve stripe 6 (*C. albipunctata*) or stripe 7 (*D. melanogaster*). See Methods in the main text for details. Click here for file
