# Supplementary material for: A quantitative atlas of Even-skipped and Hunchback expression in Clogmia albipunctata (Diptera: Psychodidae) blastoderm embryos
Source: EvoDevo. 2014 Jan 7;5:1. doi: 10.1186/2041-9139-5-1 (PMC3897886; doi:10.1186/2041-9139-5-1)
Supplement: Additional file 1: Table S1 — Polyclonal antisera against C. albipunctata Segmentation Proteins. This table summarizes the polyclonal antisera we have raised for this study. ‘Protein’ indicates the C. albipunctata gene products against which antibodies were raised. Gene nomenclature as in García-Solache and colleagues, Dev Biol 2010, 344:308–318. ‘Species’ indicates which animal was used for the immunization protocol. ‘Serum’ identifies distinct immunization reactions (two injections into different animals per gene product). ‘Rating’: ‘+++’ indicates minimal background and clear signal; ‘++’ indicates that higher concentrations of antibody are needed and background is significant; ‘+’ indicates a recognizable staining pattern, but low signal-to-noise ratio. Rating scheme equivalent to that used in Kosman and colleagues, Dev Genes Evol 1998, 208:290–294. [file 2041-9139-5-1-S1.pdf]

Table S1. Polyclonal antisera against *C. albipunctata* Segmentation Proteins.

This table summarises the polyclonal antisera we have raised for this study. ‘Protein’ indicates the *C. albipunctata* gene products against which antibodies were raised. Gene nomenclature as in García-Solache *et al.*, 2010; Dev Biol 344: 308–18. ‘Species’ indicates which animal was used for the immunisation protocol. ‘Serum’ identifies distinct immunisation reactions (two injections into different animals per gene product). ‘Rating’: ‘+++’ indicates minimal background and clear signal; ‘++’ indicates that higher concentrations of antibody are needed and background is significant; ‘+’ indicates a recognisable staining pattern, but low signal-to-noise ratio. Rating scheme equivalent to that used in Kosman *et al.*, 1998; Dev Genes Evol 208: 290–4.

Protein	Species	Serum	Rating
Hb	rabbit	SK4433	+++
		SK4434	+++
Gt	guinea pig	SKC037	+++
		SKC038	+
Knl	guinea pig	SKC039	++
		SK040	+
Eve1	guinea pig	SKC043	+
		SKC044	++
