# Supplementary material for: A quantitative atlas of Even-skipped and Hunchback expression in Clogmia albipunctata (Diptera: Psychodidae) blastoderm embryos
Source: EvoDevo. 2014 Jan 7;5:1. doi: 10.1186/2041-9139-5-1 (PMC3897886; doi:10.1186/2041-9139-5-1)
Supplement: Additional file 2: Table S2 — Number of embryo expression profiles used for data quantification, per gene and time class. [file 2041-9139-5-1-S2.pdf]

Table S2. Number of embryo expression profiles used for data quantification, per gene and time class.

	C13	T1	T2	T3	T4	T5	T6	T7	T8	Total
**Eve**	45	45	15	50	22	23	24	42	29	295
**Hb**	31	34	15	38	11	14	17	32	17	209
