# Supplementary material for: A quantitative atlas of Even-skipped and Hunchback expression in Clogmia albipunctata (Diptera: Psychodidae) blastoderm embryos
Source: EvoDevo. 2014 Jan 7;5:1. doi: 10.1186/2041-9139-5-1 (PMC3897886; doi:10.1186/2041-9139-5-1)
Supplement: Additional file 3: Table S3 — Number of embryos per time class according to physiological age. This table plots the number of embryos per assigned time class (C10-C13, C14A: T1-T8) versus the time of fixation (in hours:minutes after egg activation). See Methods in the main text for details on egg activation, embryo fixation, and assignment of embryos to time classes. [file 2041-9139-5-1-S3.pdf]

**Table S3. Number of embryos per time class according to physiological age.**
This table plots the number of embryos per assigned time class (C10–C13, C14A: T1–T8) versus the time of fixation (in hr:min after egg activation). See Materials and Methods in the main text for details on egg activation, embryo fixation, and assignment of embryos to time classes.

| | C10 | C11 | C12 | C13 | T1 | T2 | T3 | T4 | T5 | T6 | T7 | T8 | TOTAL |
|---|---|---|---|---|---|---|---|---|---|---|---|---|---|
| **3:15** | 11 | 1 | | | | | | | | | | | 12 |
| **3:30** | | 10 | | | | | | | | | | | 10 |
| **4:00** | | | 17 | | | | | | | | | | 17 |
| **4:30** | | | | 29 | | | | | | | | | 29 |
| **4:45** | | | | 4 | | | | | | | | | 4 |
| **5:30** | | | | 5 | | | | | | | | | 5 |
| **5:40** | | | | 4 | 21 | 5 | | | | | | | 30 |
| **6:00** | | | | 4 | 11 | 1 | | | | | | | 16 |
| **6:30** | | | | | 4 | | | | | | | | 4 |
| **6:45** | | | | | 1 | 12 | 22 | 1 | | | | | 36 |
| **7:00** | | | | | | 2 | 17 | 18 | 4 | | 1 | | 42 |
| **7:30** | | | | | | | 13 | 12 | 13 | 25 | 24 | 5 | 92 |
| **7:45** | | | | | | | | | 9 | 8 | 12 | 15 | 44 |
| **8:00** | | | | | | | | | | 2 | 8 | 23 | 33 |
| **Total** | 11 | 11 | 17 | 46 | 37 | 20 | 52 | 31 | 26 | 35 | 45 | 43 | 374 |
