# Supplementary material for: A quantitative atlas of Even-skipped and Hunchback expression in Clogmia albipunctata (Diptera: Psychodidae) blastoderm embryos
Source: EvoDevo. 2014 Jan 7;5:1. doi: 10.1186/2041-9139-5-1 (PMC3897886; doi:10.1186/2041-9139-5-1)
Supplement: Additional file 4: Table S4 — Average positions and shifts of Eve stripe peaks in C. albipunctata (Ca) and D. melanogaster (Dm) embryos. This table shows the position (in % position along the A-P axis, where 0% is the anterior pole) of the point of maximum intensity within each visible Eve stripe from T3 to T8. The bottom row shows the extent (in % embryo length) of anterior temporal shifts in peak position for each detectable Eve stripe, calculated from the time of stripe appearance to T8. Positions and shifts are calculated from integrated data. See Methods in the main text for details. [file 2041-9139-5-1-S4.pdf]

Table S4. Average positions and shifts of Eve stripe peaks in *C. albipunctata* (*Ca*) and *D. melanogaster* (*Dm*) embryos.
This table shows the position (in % position along the A–P axis, where 0% is the anterior pole) of the point of maximum intensity within each visible Eve stripe from T3 to T8. The bottom row shows the extent (in % embryo length) of anterior temporal shifts in peak position for each detectable Eve stripe, calculated from the time of stripe appearance to T8. Positions and shifts are calculated from integrated data. See Materials and Methods in the main text for details.

	**Stripe 1**		**Stripe 2**		**Stripe 3**		**Stripe 4**		**Stripe 5**		**Stripe 6**		**Stripe 7**	
	Ca	*Dm*	*Ca*	*Dm*	*Ca*	*Dm*	*Ca*	*Dm*	*Ca*	*Dm*	*Ca*	*Dm*	*Ca*	*Dm*
**T3**	39.18	32.38	-	42.66	-	50.84	-	59.41	-	-	-	-	-	83.68
**T4**	38.08	31.94	53.99	42.40	-	50.87	-	58.18	-	66.12	-	72.94	-	84.08
**T5**	36.11	31.65	51.67	41.68	63.98	50.05	74.42	57.34	81.71	65.12	90.34	72.45	-	82.75
**T6**	35.76	31.56	50.00	41.35	62.16	49.74	73.14	57.11	80.52	64.77	92.07	72.28	-	81.84
**T7**	35.70	31.69	48.97	40.82	60.16	49.14	70.96	56.47	78.98	63.75	90.20	71.11	-	80.26
**T8**	33.46	31.89	47.00	40.77	58.22	48.90	68.56	55.99	77.27	62.91	88.33	70.32	-	79.32
**Shift**	5.72	0.49	6.99	1.89	5.76	1.94	5.87	3.42	4.44	3.21	2.01	2.62	-	4.36
