# Supplementary material for: A quantitative atlas of Even-skipped and Hunchback expression in Clogmia albipunctata (Diptera: Psychodidae) blastoderm embryos
Source: EvoDevo. 2014 Jan 7;5:1. doi: 10.1186/2041-9139-5-1 (PMC3897886; doi:10.1186/2041-9139-5-1)
Supplement: Additional file 5: Table S5 — Width of the Eve domain in C. albipunctata and D. melanogaster embryos. Data shown for time classes T5-T8. Total domain widths (in % egg length) are calculated - from integrated data - as reaching from the peak of Eve stripe 1 to the peak of Eve stripe 6 (C. albipunctata) or stripe 7 (D. melanogaster). See Methods in the main text for details. [file 2041-9139-5-1-S5.pdf]

Table S5. Width of the Eve domain in *C. albipunctata* and *D. melanogaster* embryos.
Data shown for time classes T5–T8. Total domain widths (in % egg length) are calculated—from integrated data—as reaching from the peak of Eve stripe 1 to the peak of Eve stripe 6 (*C. albipunctata*) or stripe 7 (*D. melanogaster*). See Materials and Methods in the main text for details.

	T5		T6		T7		T8	
	Width	STDEV	Width	STDEV	Width	STDEV	Width	STDEV
C. albipunctata	54.40	1.26	56.17	2.99	54.59	2.96	54.80	4.44
D. melanogaster	51.10	6.48	50.28	6.70	48.57	4.98	47.43	4.63
